# Supplementary material for: Multi‐dataset identification of innovative feature genes and molecular mechanisms in keratoconus
Source: J Cell Mol Med. 2024 Sep 19;28(18):e70079. doi: 10.1111/jcmm.70079 (PMC11412914; doi:10.1111/jcmm.70079)
Supplement: Supplementary file 2 — Material S2. [file JCMM-28-e70079-s002.zip › 7.GSEA analysis/6.SNORA74B/KEGG_LYSINE_DEGRADATION.html]

Details for gene set KEGG\_LYSINE\_DEGRADATION[GSEA]

|  || Dataset | SNORA74B.group.cls#h\_versus\_l |
| Phenotype | group.cls#h\_versus\_l |
| Upregulated in class | l |
| GeneSet | KEGG\_LYSINE\_DEGRADATION |
| Enrichment Score (ES) | -0.41978452 |
| Normalized Enrichment Score (NES) | -1.3754067 |
| Nominal p-value | 0.17786561 |
| FDR q-value | 0.34414127 |
| FWER p-Value | 0.918 |
Table: GSEA Results Summary

  

Fig 1: Enrichment plot: KEGG\_LYSINE\_DEGRADATION      
 Profile of the Running ES Score & Positions of GeneSet Members on the Rank Ordered List

  

| SYMBOL | TITLE | RANK IN GENE LIST | RANK METRIC SCORE | RUNNING ES | CORE ENRICHMENT || 1 | OGDHL | na | 5900 | 0.196 | -0.1307 | No |
| 2 | ASH1L | na | 7848 | 0.140 | -0.1587 | No |
| 3 | BBOX1 | na | 7893 | 0.139 | -0.1321 | No |
| 4 | AASDH | na | 8463 | 0.125 | -0.1232 | No |
| 5 | TMLHE | na | 8690 | 0.120 | -0.1055 | No |
| 6 | DLST | na | 9731 | 0.098 | -0.1157 | No |
| 7 | NSD1 | na | 10685 | 0.080 | -0.1271 | No |
| 8 | ALDH3A2 | na | 13104 | 0.046 | -0.1876 | No |
| 9 | SETMAR | na | 13979 | 0.036 | -0.2055 | No |
| 10 | SETDB2 | na | 14995 | 0.024 | -0.2299 | No |
| 11 | EHMT1 | na | 15055 | 0.024 | -0.2267 | No |
| 12 | ACAT2 | na | 15497 | 0.020 | -0.2355 | No |
| 13 | ACAT1 | na | 16541 | 0.009 | -0.2638 | No |
| 14 | ECHS1 | na | 18214 | -0.008 | -0.3103 | No |
| 15 | DOT1L | na | 18875 | -0.016 | -0.3262 | No |
| 16 | HADHA | na | 19437 | -0.022 | -0.3379 | No |
| 17 | SETD1A | na | 19444 | -0.022 | -0.3335 | No |
| 18 | AASDHPPT | na | 19616 | -0.024 | -0.3336 | No |
| 19 | ALDH7A1 | na | 19848 | -0.027 | -0.3349 | No |
| 20 | SETD1B | na | 20451 | -0.034 | -0.3455 | No |
| 21 | HADH | na | 20699 | -0.036 | -0.3453 | No |
| 22 | PIPOX | na | 21698 | -0.048 | -0.3644 | No |
| 23 | OGDH | na | 23166 | -0.068 | -0.3930 | No |
| 24 | EHMT2 | na | 23481 | -0.072 | -0.3875 | No |
| 25 | SETD7 | na | 24603 | -0.092 | -0.4014 | Yes |
| 26 | SETDB1 | na | 24675 | -0.093 | -0.3848 | Yes |
| 27 | ALDH9A1 | na | 25479 | -0.107 | -0.3864 | Yes |
| 28 | SETD2 | na | 26019 | -0.117 | -0.3783 | Yes |
| 29 | GCDH | na | 26172 | -0.120 | -0.3584 | Yes |
| 30 | EHHADH | na | 26637 | -0.131 | -0.3455 | Yes |
| 31 | ALDH1B1 | na | 27039 | -0.140 | -0.3288 | Yes |
| 32 | SUV39H1 | na | 27600 | -0.154 | -0.3139 | Yes |
| 33 | PLOD3 | na | 27651 | -0.155 | -0.2841 | Yes |
| 34 | SUV39H2 | na | 29151 | -0.199 | -0.2872 | Yes |
| 35 | PLOD2 | na | 31313 | -0.284 | -0.2923 | Yes |
| 36 | PLOD1 | na | 31772 | -0.311 | -0.2430 | Yes |
| 37 | AADAT | na | 32052 | -0.325 | -0.1855 | Yes |
| 38 | ALDH2 | na | 34359 | -0.636 | -0.1240 | Yes |
| 39 | AASS | na | 34441 | -0.668 | 0.0083 | Yes |
Table: GSEA details [plain text format]

  

Fig 2: KEGG\_LYSINE\_DEGRADATION      
 Blue-Pink O' Gram in the Space of the Analyzed GeneSet

  

Fig 3: KEGG\_LYSINE\_DEGRADATION: Random ES distribution      
 Gene set null distribution of ES for **KEGG\_LYSINE\_DEGRADATION**
